# Serpin peptidase inhibitor, clade E, member 2 in physiology and pathology: recent advancements

**DOI:** 10.3389/fmolb.2024.1334931

**Published:** 2024-02-26

**Authors:** Shutong Wu, Yuchao Yang, Meiling Zhang, Asmat Ullah Khan, Jingxing Dai, Jun Ouyang

**Affiliations:** ^1^ Guangdong Provincial Key Laboratory of Digital Medicine and Biomechanics, Guangdong Engineering Research Center for Translation of Medical 3D Printing Application, National Virtual & Reality Experimental Education Center for Medical Morphology (Southern Medical University), National Key Discipline of Human Anatomy, School of Basic Medical Sciences, Southern Medical University, Guangzhou, China; ^2^ Xinjin Branch of Chengdu Municipal Public Security Bureau, Chengdu, China; ^3^ Yue Bei People’s Hospital Postdoctoral Innovation Practice Base, Southern Medical University, Guangzhou, China; ^4^ Chengdu Municipal Public Security Bureau Wenjiang Branch, Chengdu, China

**Keywords:** conformational diseases, hemostasis, osteoarthritis, pathology, physiological processes, reproductive, serpin E2, tumor metastasis

## Abstract

Serine protease inhibitors (serpins) are the most numerous and widespread multifunctional protease inhibitor superfamily and are expressed by all eukaryotes. Serpin E2 (serpin peptidase inhibitor, clade E, member 2), a member of the serine protease inhibitor superfamily is a potent endogenous thrombin inhibitor, mainly found in the extracellular matrix and platelets, and expressed in numerous organs and secreted by many cell types. The multiple functions of serpin E2 are mainly mediated through regulating urokinase-type plasminogen activator (uPA, also known as PLAU), tissue-type plasminogen activator (tPA, also known as PLAT), and matrix metalloproteinase activity, and include hemostasis, cell adhesion, and promotion of tumor metastasis. The importance serpin E2 is clear from its involvement in numerous physiological and pathological processes. In this review, we summarize the structural characteristics of the Serpin E2 gene and protein, as well as its roles physiology and disease.

## Introduction

Serpin peptidase inhibitor, clade E, member 2 (serpin E2) is considered an extracellular matrix (ECM) protein, but can also be distributed in the cell membrane and cytoplasm. The molecular weight of the serpin E2 protein is in the range 45–50 kDa, and it is encoded by a gene on human chromosome 2q99-q35 (Sommer et al., 1987). Serpin E2 belongs to the nexin protease family, and it is also known as protease nexin-1 (PN-1) (Yang et al., 2018), which is highly expressed in the human brain, platelets, gonads, and bone (Crisp et al., 2000).

Serpin E2 was initially reported in 1978, after its detection in glial cell culture media as a neurite-promoting factor (Barde et al., 1978). The expression of serpin E2 is broadly distributed throughout the brain, and particularly strong in the hippocampus and amygdala. Serpin E2 can regulate neuronal activity, and the characteristics of synapses between neurons are influenced by its expression levels (Lüthi et al., 1997; Meins et al., 2010). Further, Serpin E2 is associated with fear regulation and regression (Meins et al., 2010), epilepsy occurrence (Lüthi et al., 1997), and prevention of cerebral ischemia in mice (Mirante et al., 2013).

A wide range of signaling pathways involving Serpin E2 have significant roles in the development of various diseases. Serpin E2 is a crucial biomarker for the diagnosis and prognosis of various malignancies, and recent research has focused on its function in the development of diverse tumors (Gao et al., 2008; Nagahara et al., 2010; Zheng et al., 2013.). Moreover, deletion or overexpression of Serpin E2 is frequently associated with the occurrence or progression of various diseases (François et al., 2018; Li L. et al., 2019).

In this article, we briefly review the known functions of serpin E2 and introduce its roles in physiological and pathological processes, as well as the signaling pathways involved.

## Serpin E2 expression and structure

All serpins have the same tertiary structure, which includes three β folds (A, B, C) and nine α helices (hA–hI) (Law et al., 2006). Serpins bind to the active sites of their target proteins through an exposed reactive center loop (RCL), which, in contrast to other inhibitors, is mobile and can freely enter and exit Aβ folding conformation (Hopkins et al., 1993). When target proteases bind to serpins in an intermediate, metastable state (instead of their most stable conformation) in the cell, they cleave the reaction center of serpins, leading to rapid mobility of the RCL, which forces target protease binding to serpins (Carrell and Lomas, 1997; Law et al., 2006). Since they mediate irreversible inhibition, serpins are considered primary inhibitors of intracellular and extracellular proteolysis pathways (Huntington et al., 2000).

Among the serpins, serpin E2 is a powerful alkaline protein that has the same secondary structure as other serpins. Serpin E2 also follows the classical folding structure of serpin family; however, the helix direction in serpin E2 is different from that of other serpins. Further, unlike other serpins, serpin E2 is neither synthesized in the liver nor does it circulate in the blood, and it is barely detectable in plasma (Li and Huntington, 2012). Nevertheless, serpin E2 is expressed in multiple organs and by various cell types, including macrophages, astrocytes, smooth muscle cells, vascular cells, and platelets (Choi et al., 1990; Bouton et al., 2003; 2012; Mansilla et al., 2008; Li and Huntington, 2012).

Many serpin E2 functions are related to its inhibitory effect on plasminogen activator (PA), including regulation of hemostasis, cell adhesion, and promotion of tumor metastasis (Czekay and Loskutoff, 2009; Bharadwaj et al., 2021; Bianchini et al., 2021). There are two types of PA in humans: uPA and tPA (Danø et al., 1985; Vassalli et al., 1991).

## Serpin E2 in Physiological Processes

### Serpin E2 plays a significant role in the nervous system

Serpin E2 is thought to regulate neurite outgrowth in the adult nervous system. The timing and rate of neurite outgrowth are critical for neuronal cell differentiation, which is linked to maturation of the nervous system (Monard, 1988). Schwann cells respond to injury by proliferating and differentiating into myelinating cells, which form the myelin sheaths of myelinated nerve fiber axons in the peripheral nervous system (Kioussi and Gruss, 1996). In rats, serpin E2 mRNA and protein in Schwann cells increases momentarily and significantly after sciatic nerve injury at the distal end of the injury site, while only modest amounts of serpin E2 are detectable in intact rat sciatic nerves (Meier et al., 1989; Kioussi and Gruss, 1996). Serpin E2 levels increase by 5–6 times during *in vitro* simulated sciatic nerve injury, compared with before the injury. Moreover, serpin E2 expression levels are negatively correlated with those of angiotensin Ⅱ receptor subtype AT_1_. It was speculated that nerve injury reduces AT_1_ expression, resulting in a sharp increase of serpin E2 (Bleuel and Monard, 1995). Overall, there is strong evidence that serpin E2 has an important role in myelinated nerve fiber axonal injury repair.

Sonic hedgehog (SHH) is a morphogenetic protein with a crucial role in vertebrate organ development regulation, and the primary signal for proliferation of cerebellar granular neuron precursors (CGNPs) (Kenney and Rowitch, 2000, 1; Sagai et al., 2019). In animal studies, serpin E2 inhibited SHH-induced CGNP proliferation during mouse cerebellum development, reducing the ensuing rise in mature cerebellar cells. Hence, the interaction between serpin E2 and SHH is very important for mouse cerebellar development (Vaillant et al., 2007). In another study on lzheimer’s mice (AD mice), it was mentioned that PN-1 can inhibit the activation of SHH pathway in AD mice to affect the development of Alzheimer‘s disease in mice (Li et al., 2021). SHH can also stimulate hippocampal neural stem cell proliferation, and regulates the differentiation of hippocampal neural progenitor cells into neurons (Lai et al., 2003).

As a thrombin inhibitor, serpin E2 is consider crucial for inhibiting thrombin-mediated ischemic neuronal death in the central nervous system (de Castro Ribeiro et al., 2006). In addition to the hippocampus, serpin E2 defects also alter the amygdala, which is responsible for emotional responses (such as fear, anxiety, *etc.*) and memory processing (Ferry et al., 1999; Feinstein et al., 2011). Fos protein is considered a marker of fear extinction, and in situations that promote fear extinction, Fos immunoreactivity in the basal ganglia of wild-type (WT) mice is considerably higher than that in serpin E2-defective mice (Meins et al., 2010). Thus, serpin E2 represents a potential new target for treatment in a range of anxiety and stress-related diseases.

### Serpin E2 plays an anticoagulant role in hemostasis

Hemostasis is a complex physiological process. In general, rapid thrombus formation is typically necessary to stop the bleeding after vascular injury, and the thrombus cannot affect the blood flow through the blood vessel (Leslie, 2010; Garmo et al., 2022). Serpin E2, a member of the serine protease inhibitors superfamily, is a part of the humoral anticoagulation system (Garmo et al., 2022), which can inhibit multiple serine proteases, including trypsin, thrombin, and activated protein C (Bouton et al., 2012). Serpin E2 binds to glycosaminoglycans on the surface of platelets in blood, and is stored in platelet α granules (Boulaftali et al., 2010, 1), and on binding to glycosaminoglycan, serpin E2 inhibits thrombin (Richard et al., 2006).

Serpin E2 can be detected in resting platelet extracts after washing to separate them, while platelet secretion products (i.e., the supernatant) lack thrombin inhibition activity and serpin E2. After treatment with a strong platelet agonist in the resting state, serpin E2 cannot be detected in platelet extract but is found in platelet secretion products, and the results obtained after treatment with weak platelet agonists are opposite to those obtained with strong platelet agonist. Further, when activated platelet secretory products and thrombin are combined, thrombin is almost completely inhibited; however, this inhibitory effect disappears when serpin E2 blocking antibody is added (Boulaftali et al., 2010, 1). These data indicate that serpin E2 is secreted during platelet activation and act as a thrombin inhibitor after its secretion.

Fucoidan and low and high molecular weight (HMW) heparin both effect serpin E2 activity and distribution (Richard et al., 2006). Fucoidan, a molecule extracted from brown algae, has a various biological functions (Fitton et al., 2015), including as an anticoagulant, antithrombotic (Li B. et al., 2019), anti-tumor, and anti-inflammatory factor (Takahashi et al., 2017). Unfractionated heparin and HMW fucoidan can accelerate the inhibitory effect of serpin E2 on thrombin by creating a ternary complex. The anticoagulant effect of serpin E2 is more potent than that of antithrombin (AT), and some *in vitro* studies have shown that serpin E2 inhibits thrombin approximately 100 times more quickly than AT (Wallace et al., 1989). Further, the anticoagulant effect of serpin E2 is stronger than that of AT regardless of whether or not heparin is present. The powerful anticoagulant function of serpin E2 makes it a protect factor for venous thromboembolism (Li et al., 2023). In addition, due to its anticoagulant properties, blocking serpin E2 can improve coagulation dysfunction in patients with mild to moderate hemophilia (Aymonnier et al., 2019; 2021).

The role of serpin E2 as an endogenous thrombin inhibitor in vascular physiology is an underappreciated though the three-dimensional structure of serpin E2 and its interaction mechanism with heparin have been reported (Bouton et al., 2012; Li and Huntington, 2012). Nevertheless, targeted therapy for serpin E2 is predicted to become more common in clinical practice as a result of research into its anticoagulant properties.

### The role of serpin E2 in angiogenesis

Angiogenesis is the development of new blood vessels from existing blood vessels, due to endothelial cell proliferation and migration. Various signal transduction pathways are involved in angiogenesis, which is regulated by numerous factors. Angiogenesis can also occur in adults under certain physiological conditions, such as wound healing or in the menstrual endometrium, while angiogenesis in specific disease states often indicates negative consequences (Selbonne et al., 2015; Apte et al., 2019). Serpin E2-deficient mice have more arteries/veins in their retinas and more muscle blood vessels than WT mice (Selbonne et al., 2015). Further, Matrigel embolization assays also showed that there was more angiogenesis in emboli from serpin E2-deficient mice (Selbonne et al., 2012).

In a study on skeletal muscle ischemia in mice, serpin E2-deficient mice had a faster rate of femoral artery reperfusion after femoral artery ligation to create hindlimb ischemia than WT mice. Capillary density was then analyzed in the two different mouse strains and neovascularization was stronger in serpin E2-deficient mice than that in WT mice, indicating that lack of serpin E2 was beneficial to blood flow reperfusion in ischemic muscles. Lack of serpin E2, resulting in angiogenesis, may be responsible for the observed rise in MCP-1(Monocyte chemoattractant protein-1) level and increased leukocyte recruitment (Selbonne et al., 2021).

The role of serpin E2 in vascular biology was not investigated until the last decade. Various studies have demonstrated that serpin E2 has an anti-angiogenesis role, because it interacts with numerous molecules involved in angiogenesis, including VEGF, MCP-1, and Smad5, among others (Selbonne et al., 2015; Apte et al., 2019; Madjene et al., 2021). By restricting the growth of new blood vessels under pathological conditions, serpin E2 plays an essential role in maintaining angiogenesis homeostasis under physiological conditions in adults and in healthy human physiology.

### The role of serpin E2 in reproductive processes

Serpin E2 is widely expressed in the placenta and uterus in both humans and mice (White et al., 1993; Hofmann et al., 1994; Teesalu et al., 1998), and plays an important role in reproductive processes, being associated with breastfeeding, pregnancy, and the estrous cycle in mice (Chern et al., 2010; 2011; Liu et al., 2023). In immature human oocytes, high serpin E2 levels can downregulate hyaluronan synthase 2 and versican expression by binding to uPA in cumulus cell ECM, reducing the hyaluronic acid content of the matrix. Additionally, serpin E2 inhibits cumulus expansion and hinders oocyte maturation, and exogenous serpin E2 can significantly reduce oocyte maturity; however, in mature oocytes, serpin E2 expression was considerably lower than that in immature oocytes, and serpin E silencing or overexpression had little effect on cumulus-oocyte complexes (Lu et al., 2013). Serpin E2 was also found to be highly expressed in the apical and glandular epithelium of the endometrial lumen in the middle and late secretory phases of the menstrual cycle, but weakly expressed in the proliferative phase of the endometrium. Further, serpin E2 is thought to play a role in embryo implantation, because of its high expression during the secretory phase and can regulate the proteolytic degradation and fibrinolysis of extracellular matrix in endometrial cells, which is crucial for decidualization and trophoblast invasion during implantation, and its function in the human uterus may be related to tissue remodeling regulated by PA (Lee et al., 2011; AbdelHafez et al., 2023; Do et al., 2023).

**FIGURE 1:**
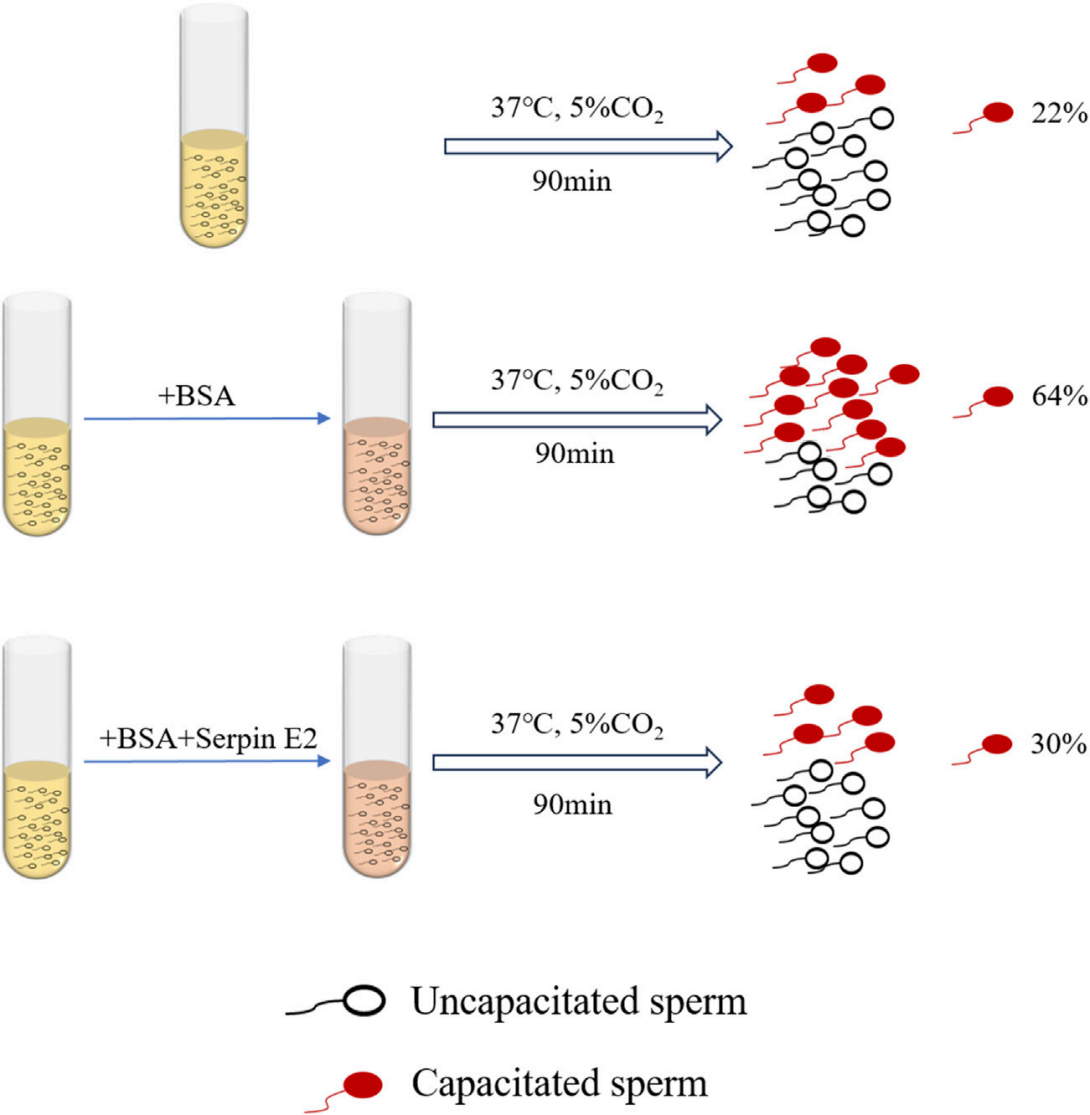
Effects of Serpin E2 on murine sperm capacitation.

Serpin E2 affects decapacitation factors during sperm capacitation (Lu et al., 2011; Guidobaldi et al., 2017; Li et al., 2018). Sperm from the tail of the epididymis of male mice were divided into two groups: one incubated with BSA only for capacitation, and the other with BSA followed by serpin E2. After treatment, sperm capacitation was observed and samples treated with serpin E2 had significantly inhibited sperm capacitation compared with control group sperm treated with BSA alone (Figure 1) (Lu et al., 2011). Further capacitated sperm in the oviduct of female mice were treated with serpin E2 3 h after mating, then A23187 ionophore applied to induce the acrosome reaction. The acrosome response in the experimental group was significantly inhibited compared to the control group (without serpin E2 treatment) (Li et al., 2018). These findings suggest that serpin E2 can reversibly regulate mouse sperm from capacitation to non-capacitation both *in vivo* and *in vitro*.

Sperm activity can be regulated by the PA molecules, tPA and uPA, which are widely distributed in the male reproductive tract (Gunnarsson et al., 1999; Martinez-Soto et al., 2018). Further, male fertility can be reduced by plasminogen-fibrinolytic system disorder (Ebisch et al., 2007). According to some studies, oocytes release PA when sperm bind to the oocyte extracellular layer via surface receptors, impairing the ability of sperm to adhere and reducing human fertility. As a serine protease inhibitor, serpin E2, can affect sperm motility by regulating the activities of tPA, uPA, and other serine proteases in semen (Coy et al., 2012). Deletion of the serpin E2 gene can lead to infertility in male mice (Murer et al., 2001, 1); therefore, plasminogen-plasmin system homeostasis in semen is critical (Maier et al., 1991; He et al., 1999). This principle can also be used for contraception and assisted reproductive technology, and future breakthroughs are expected (Table 1).

**TABLE 1:** Serpin E2 in physiological processes.

Signaling pathway	Physiological function	References
AT1	Repair damaged myelinated nerve fiber axonal	Bleuel and Monard (1995)
SHH	Mouse cerebellar development	Vaillant et al. (2007)
Fos	Promote fear extinction	(Meins et al., 2010)a
Glycosaminoglycan	Anticoagulation	Richard et al. (2006)
MCP-1	Anti-angiogenesis	Selbonne et al. (2021)
uPA	Reduce oocyte maturity	Lu et al. (2013)
Plasminogen-fibrinolytic system	Decrease sperm motility	Murer et al. (2001), Coy et al. (2012)

## Roles of serpin E2 in pathological processes

### Effect of serpin E2 on osteoarthritis

Osteoarthritis (OA), the most common rheumatic disease, is a degenerative condition that damages articular cartilage and the synovium, ligaments, and tendons around the joints (Santoro et al., 2015; Gratal et al., 2022; Wu et al., 2022). The knee is the joint most frequently affected by OA, followed by the finger and hip joints (Favero et al., 2022). Cartilage tissue is composed of abundant ECM and cells, mainly chondrocytes, but has no blood vessels or innervation. Under normal circumstances, chondrocytes are very stable cells that can maintain ECM stability by balancing the synthesis and degradation of some matrices; however, this balance is disturbed during OA, and pro-inflammatory cytokines (such as IL-1α and IL-1β, among others) induce expression of matrix metalloproteinase (MMP) molecules in chondrocyte matrix, resulting in ECM degradation (Goldring and Marcu, 2009; Santoro et al., 2015).

**FIGURE 2:**
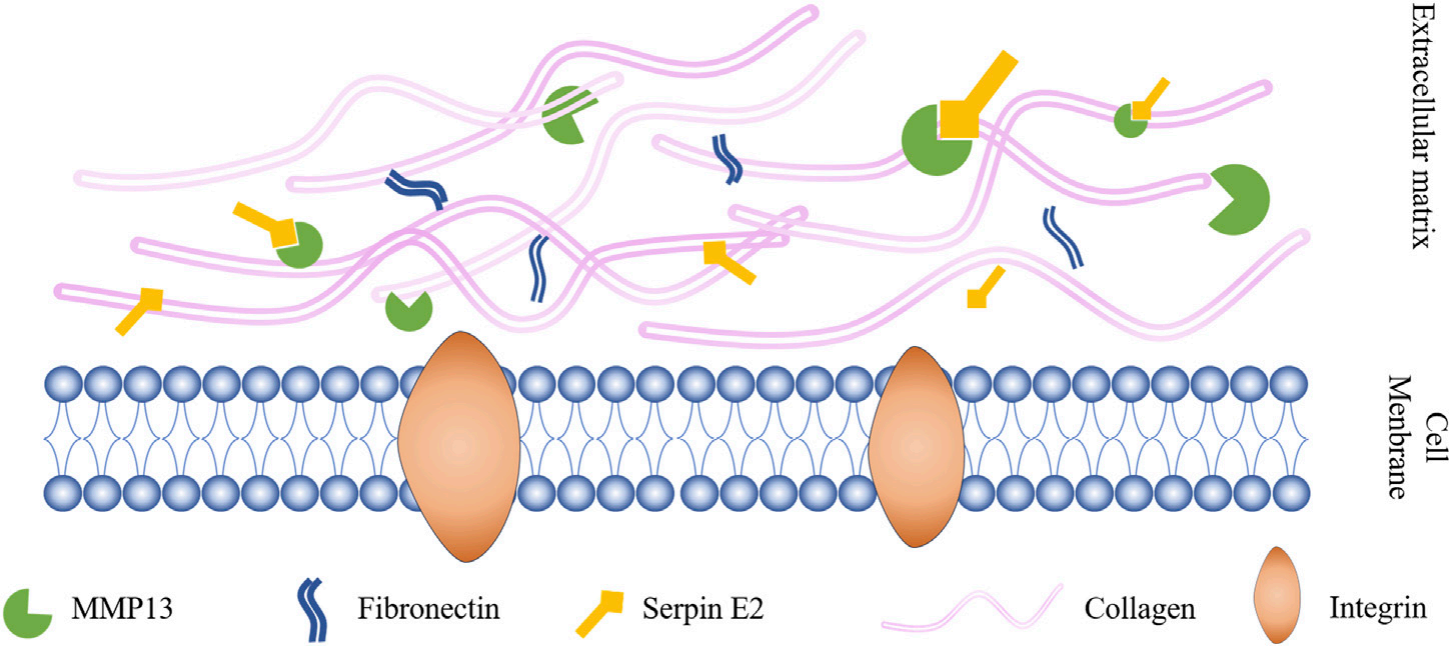
Serpin E2 improves OA by inhibiting MMP13 activity.

Interactions between serpin superfamily proteins and MMPs are crucial in OA occurrence. MMP activity in synovial fluid from patients with OA is increased compared with that in healthy people, leading to the inactivation of serpins (Jones et al., 1998), including serpin C1 (Jones et al., 1998) (also known as AT III), serpin G1 (Sanrattana et al., 2019), serpin E1 (Sadowski and Steinmeyer, 2001; Freeberg et al., 2018), and serpin E2 (Sadowski and Steinmeyer, 2001), among others. Serpin E1 is the serpin with the most similar sequence to that of serpin E2, with 41% homology between them (Sommer et al., 1987). MMP13 is among the collagenases that are primarily associated with progressive cartilage degradation in patients with OA. In exploring the mechanism by which recombinant serpin E2 inhibits MMP13 and the downstream transcription factor, NF-κB, researchers identified a pathway related to MMP13 that can trigger OA (Figure 2) (Santoro et al., 2015). Serpin E2 has also been found to inhibit cartilage degradation in rabbits (Stevens et al., 1993). In addition, Shen et al. (Shen et al., 2019) confirmed *circSERPINE2* as a key circRNA involved in OA downregulation by deep sequencing circRNA molecules expressed in OA and control cartilage tissue in *vitro* and *in vivo* experiments.

There are currently no effective drugs available to treat OA in clinical practice. Since this disease is chronic and degenerative, it causes considerable physical and psychological harm to patients. Over the last decade, research into OA pathogenesis has become increasingly in-depth, and serpin E2 has been discovered to play a prominent role, making it a promising target for clinical therapy of OA in the near future (Maciejewska-Rodrigues et al., 2010).

### High expression of serpin E2 affects tumor occurrence and development

Serpin E2 is expressed in normal cells and plays a vital role in many tumors. Moreover, serpin E2 is overexpressed in many cancers, including liver cancer (Hou et al., 2019; Zhang X. et al., 2020), non-small cell lung cancer (Dokuni et al., 2020; Zhang et al., 2022), breast cancer (Tang et al., 2019), osteosarcoma (Mao and Wang, 2016), bladder cancer (Li F. et al., 2020), and melanoma (Perego et al., 2018), among others; this overexpression can promote tumor proliferation, metastasis, and colony formation (Liu et al., 2019), which are characteristics related to the tumor microenvironment (TME) (Bejarano et al., 2021).

The TME is created by interactions between a tumor and tumor stroma, which contains stromal cells, ECM, various secreted proteins, and exosomes, among other factors (Bharadwaj et al., 2021). The TME determines the occurrence, development, and prognosis of tumors. Tumor stem cells, including those from lung (Yan et al., 2022) and breast (Tang et al., 2019) cancers, can differentiate into vascular endothelial cells to form new blood vessels. In addition, there are many sources for tumor vascular remodeling, including bone marrow-derived endothelial progenitor cells that integrate into newly formed blood vessels or tumor cell angiogenesis simulation (Weis and Cheresh, 2011). A study on oral squamous cell carcinoma (OSCC) found that serpin E2 increases angiogenesis and lymphangiogenesis in OSCC by binding to LEM domain containing 1, a factor that promotes OSCC development (Sasahira et al., 2021). In addition, serpin E2 is a factor in vascular remodeling and is crucial for the invasion and metastasis of other tumors (Wagenblast et al., 2015).

Breast cancer is among the most common malignant tumors in women. Most patients die from breast cancer metastasis, rather than from primary tumors (Tang et al., 2019). During breast cancer occurrence and development, serpin E2 binds to its target protein, uPA, through a covalent bond, to form a serpin E2/uPA complex. Further, uPA and tPA overexpression during tumorigenesis typically indicates tumor metastasis and poor prognosis (Fayard et al., 2009; Duffy et al., 2014; Jevrić et al., 2019). The serpin E2/uPA covalent complex is decomposed after binding to low-density lipoprotein receptor-related protein 1 (LRP-1), stimulating ERK activation, promoting MMP9 expression, and aggravating tumor metastasis. Animal studies indicated that serpin E2 is unnecessary for breast tumor growth, but essential for tumor metastasis, and MMP9 is a key mediator of serpin E2-mediated tumor metastasis (Buchholz et al., 2003; Fayard et al., 2009). MCF-7 cells, which are rich in breast cancer stem cells, were treated with EGF, after injection of sh-serpin E2 lentivirus into the tail vein of mice. After 40 days, mice were sacrificed, and samples collected. Hematoxylin and eosin staining showed that, compared with the EGF-negative control group, the number and size of tumors in lung and liver tissues from the transfection group animals were decreased. The effect of serpin E2 breast cancer cell metastasis was induced by EGF, as evidenced by the fact that the number of lung and liver tumors in the transfection group was significantly higher than that in the negative control group after injection of MCF-7 cells overexpressing serpin E2 (Tang et al., 2019). In addition, some studies have found that *circ*SERPINE2 acts as a communication medium in TME, mediating the positive feedback loop between tumor cells and tumor-associated macrophages, increasing the infiltration of tumor-associated macrophages and promoting the progression of breast cancer *in vivo*. These findings provide new strategies for nanotherapy of breast cancer (Zhou et al., 2023).

Serpin E2 binds directly to B cell receptor-associated protein 31 (BAP31) and is regulated by BAP31 in Hep3b and MHCC97h cells, thereby affecting ERK1/2 and p38, and controlling hepatocellular carcinoma cell proliferation and colony formation (Zhang X. et al., 2020). Serpin E2 overexpression in tumor cells also affects cancer treatment and prognosis. In radiotherapy for non-small cell lung cancer (NSCLC), DNA damage repair of tumor cells occurs through the MRN-SERPINE2-ATM-RAD51 pathway, resulting in radioresistance, leading to NSCLC radiotherapy failure (Hou et al., 2019). In 2020, Dokuni et al. (2020) collected surgical specimens from 74 patients undergoing complete surgical resection of lung adenocarcinoma. Serpin E2 expression was detected in cancer cells from 19 (26%) of the patients, and all 19 patients had metastatic lung adenocarcinoma. Overall survival curve analysis showed that high serpin E2 expression was associated with lower overall survival after lung adenocarcinoma surgery. Serpin E2 is now recognized as a marker of aggression (Perego et al., 2018; Tang et al., 2019) and prognosis (Wang et al., 2015; Langer et al., 2018) in multiple tumors.

According to a study on osteosarcoma, high serpin E2 expression can lead to drug resistance of osteosarcoma cells, as well as accelerated cell proliferation and reduced patient survival rates (Mao and Wang, 2016). Serpin E2 promotes tumor metastasis in esophageal squamous cell carcinoma by activating the bone morphogenetic protein, BMP4 (Zhang J. et al., 2020). In a study on glioma cells, it was found that *circ*SERPINE2 can inhibit the apoptosis of glioma cells and promote the proliferation, migration and invasion of glioma (Li and Lan, 2021). These studies suggest that serpin E2 can have a positive regulatory role in tumor invasion and metastasis; however, another study showed that, in prostate cancer, serpin E2 can reduce angiogenesis and block prostate cancer development by inhibiting the Hedgehog pathway (McKee et al., 2012). The results of earlier investigations of cancer stem cells are inconsistent with the role of serpin E2 in angiogenesis.

There are two main theories for the mechanism by which serpin E2 enhances tumor cell metastatic ability: 1) formation of covalent complexes by binding to its target protein, which promotes the expression of MMPs, thus degrading the ECM and mediating tumor cell metastasis; and 2) promotion of angiogenesis in the TME, leading to tumor metastasis. Tumor metastasis is a complex process involving the activation of numerous signaling pathways and formation of protein complexes. Serpin E2 has emerged as a marker of tumor prognosis, and increasing numbers of studies have investigated the mechanisms by which serpin E2 mediates tumor metastasis and its therapeutic targets (Bergeron et al., 2010), which has profound significance for the recovery of patients, prevention and treatment of tumor recurrence, and improvement of patient survival (Table 2).

**TABLE 2:** Interactions of signaling pathways with serpin E2 in tumors.

Signaling pathway	Disease	References
MAPK	HCC	Zhang et al. (2020c)
EGF/MEK/ERK	NSCLC	Dokuni et al. (2020), Zhang et al. (2022)
MRN/ATM		
EGF/PKC/MAPK/EGR1	Breast cancer	Tang et al. (2019)
CDK4	Osteosarcoma	Mao and Wang (2016)
Wnt/β-catenin	Esophageal squamous cell carcinoma	Zhang et al. (2020b)
Hedgehog	Prostate adenocarcinoma	McKee et al. (2012)

### Problems and perspectives

The role of serpin E2 in maintaining normal human physiological functions has been widely studied, and the main focus of research into its roles in various diseases has been on interactions between serpin E2 and other proteins in the ECM (Donovan et al., 1994; Cao et al., 2004).

As a serine protease inhibitor superfamily member, serpin E2 can protect articular cartilage and prevent OA by inhibiting serine proteases in the ECM, thereby inhibiting MMP activation. In contrast, serpin E2 forms a complex in tumors by binding to its target proteases, such as tPA. A “paradox” was reported in which this complex promotes MMP activity and induces tumor metastasis. A similar phenomenon was also described in a study of the serpin E2 homolog, serpin E1 (Binder and Mihaly, 2008). It has been speculated that this paradox is related to the TME (Smirnova et al., 2016). Serpin E2 has also been shown to activate ERK1/2 and β-catenin signaling pathways by interacting with LRP-1 and uPAR, which in turn promotes collagen production in fibroblasts and induces cardiac fibrosis (Li et al., 2022; Kmiotek-Wasylewska et al., 2023). However, disease occurrence and development involves complex processes, and there may be many protein changes or protein interactions that have yet to be discovered. Overall, the mechanism by which serpin E2 plays opposing roles in different diseases has yet to be elucidated, and further research is required.

Although serpin E2 is an ECM protein, it can also affect the expression of intracellular proteins. Serpin E2 can regulate the transcription of Wnt/β-catenin-independent target genes by regulating the chromatin-related APC protein, which in turn affects the prognosis of patients with colorectal cancer. Serpin E2 expression levels can predict the progression stage of colorectal cancer, but are not related to survival time in patients with this type of malignancy. Therefore, research into the effect of serpin E2 on intracellular protein expression may be of great significance and inform future discoveries (Hankey et al., 2018). In addition to OA, serpin E2 is associated with various other cartilage/bone-related diseases. For example, serpin E2 expression is reduced in degenerative disc diseases (Wu et al., 2016; Francisco et al., 2023) and autosomal dominant osteosclerosis type II (ADO II). ADO II is characterized by insufficient osteoclast activity; serpin E2 is considered to serve a compensatory function in reducing osteoclast absorption in ADO II (Del Fattore et al., 2008; Coudert et al., 2014). Femoral head necrosis (FHN) is a hip joint disease (Hopkins and Genant, 2020), and serpin E2 overexpression can alleviate FHN caused by steroid hormones by increasing osteoblast activity and reducing apoptosis (Yang et al., 2021). Hence, serpin E2 also has potential to contribute greatly to regenerative medicine, and this possibility warrants further discussion and exploration.

Abnormalities occurring during transformation of a protein three-dimensional structure can cause disorders including bovine spongiform encephalopathy (Aguzzi, 2006), Alzheimer’s (Mager et al., 2002), Parkinson’s (Watanabe et al., 2020), and Huntington’s (Tran and Miller, 1999) diseases, among others. During serpin binding, a target protease will move from the upper pole of the serpin molecule to the lower, and an extra chain is inserted into the A β-sheet (Huntington et al., 2000). Hence the inhibitory function of serpins is accompanied by conformational changes to the protease. The protein structure of serpins is relatively conserved and point mutations in serpin molecules can induce conformational diseases. Mutations in the Z allele of serpin A1 (e.g., E342K) cause its misfolding and aggregation, leading to cirrhosis and chronic obstructive pulmonary disease (Jagger et al., 2020). Further, a novel heterozygous missense mutation of serpin C1 can lead to type I hereditary AT deficiency, resulting in venous thromboembolism or severe dementia due, to abnormal polymer retention in cells (Zhang F. et al., 2020). In addition, mutation of serpin I1 (also known as neuroserpin) can cause familial encephalopathy with neuroserpin inclusion bodies (Davis et al., 1999; 2002; Crowther, 2002). There are no reports of conformational disease caused by mutation of serpin E2 but, given the structural characteristics of the superfamily, related research findings are likely in the near future.

COVID-19 has spread worldwide and claimed millions of lives to date. The degree of disease progression in patients with COVID-19 is related to many factors, including age, the presence of underlying disease, and genetic susceptibility (Li X. et al., 2020). Serpin E2 inhibits the activity of furin protein and plasmin, and in drug treatment for COVID-19, serpin E2 expression is increased using a mineralocorticosteroid receptor antagonist, which reduces plasmin and furin protein activity, thereby inhibiting proteolysis, binding of COVID-19 to ACE2, and reducing viral infectivity (Wilcox and Pitt, 2020).

In addition, a genome-wide association study and bioinformatics analysis showed that serpin E2 gene polymorphism is associated with susceptibility to chronic obstructive pulmonary disease; however, the underlying mechanism has not been determined (Li L. et al., 2019; Paci et al., 2020).

Overall, serpin E2 has an essential role in maintaining normal physiological processes. Research on serpin E2 in pathological processes to date has focused on tumors, due to its widespread distribution and complex function; however, it is also closely associated with the occurrence and development of many other diseases and warrants further study in these contexts.

## Abbreviations

serpins, serine protease inhibitors; serpin E2, serpin peptidase inhibitor, clade E, member 2; ECM, extracellular matrix; uPA, urokinase-type plasminogen activator; tPA, tissue-type plasminogen activator; MMP, matrix metalloproteinase; PN-1, Protease nexin-1; RCL, reactive center loop; PA, plasminogen activator; AngⅡ, angiotensin Ⅱ; SHH, sonic hedgehog; CGNPs, cerebellar granular neuron precursors; LMW, low molecular weight; HMW, high molecular weight; AT, antithrombin; MCP-1, Monocyte chemoattractant protein-1; OA, osteoarthritis; TME, tumor microenvironment; OSCC, oral squamous cell carcinoma; LRP-1, low-density lipoprotein receptor-related protein 1; BAP31, B cell receptor-associated protein 31; HCC, hepatocellular carcinoma; NSCLC, non-small cell lung cancer; ADO II, autosomal dominant osteosclerosis type II; FHN, femoral head necrosis.
